# Measuring Quality Gaps in TB Screening in South Africa Using Standardised Patient Analysis

**DOI:** 10.3390/ijerph15040729

**Published:** 2018-04-12

**Authors:** Carmen S. Christian, Ulf-G. Gerdtham, Dumisani Hompashe, Anja Smith, Ronelle Burger

**Affiliations:** 1 Department of Economics, University of the Western Cape, Bellville 7535, South Africa; 2 Department of Economics, Stellenbosch University, Stellenbosch 7602, South Africa; dhompashe@ufh.ac.za (D.H.); anja.smith1@gmail.com (A.S.); rburger@sun.ac.za (R.B.); 3 Department of Economics, Lund University, SE-220 07 Lund, Sweden; ulf.gerdtham@med.lu.se; 4 Department of Clinical Science (Malmo), Lund University, SE-202 13 Malmö, Sweden; 5 Department of Economics, University of Fort Hare, Alice 5700, South Africa

**Keywords:** quality of care, quality gaps, TB screening, standardised patients, South Africa

## Abstract

This is the first multi-district Standardised Patient (SP) study in South Africa. It measures the quality of TB screening at primary healthcare (PHC) facilities. We hypothesise that TB screening protocols and best practices are poorly adhered to at the PHC level. The SP method allows researchers to observe how healthcare providers identify, test and advise presumptive TB patients, and whether this aligns with clinical protocols and best practice. The study was conducted at PHC facilities in two provinces and 143 interactions at 39 facilities were analysed. Only 43% of interactions resulted in SPs receiving a TB sputum test and being offered an HIV test. TB sputum tests were conducted routinely (84%) while HIV tests were offered less frequently (47%). Nurses frequently neglected to ask SPs whether their household contacts had confirmed TB (54%). Antibiotics were prescribed without taking temperatures in 8% of cases. The importance of returning to the facility to receive TB test results was only explained in 28%. The SP method has highlighted gaps in clinical practice, signalling missed opportunities. Early detection of sub-optimal TB care is instrumental in decreasing TB-related morbidity and mortality. The findings provide the rationale for further quality improvement work in TB management.

## 1. Introduction

Developing countries in sub-Saharan Africa and Asia account for close to 80% of the global TB burden [1]. It is therefore unsurprising that South Africa, a high-burden TB country, struggles with this pandemic which remains the main driver of morbidity and mortality [2]. Within this context, local and international research highlighting gaps and weaknesses in TB protocol implementation [3,4,5,6,7,8] and a global call for shifts towards quality improvement [9,10,11] motivated us to consider the quality of TB screening in South Africa.

There is growing consensus that increasing healthcare coverage is unlikely to improve health outcomes if the quality of care is lagging [12,13,14,15]. There are few studies on the quality of TB diagnosis and treatment [11]. Some of these use novel approaches yet still find that there are significant gaps in the quality of TB care in high-burden countries [7]. Our study adds to the literature in the quality of care space.

Measuring quality of care comes with challenges [16,17]. The available measurement tools have strengths and weaknesses. At a facility level, few reliable measures exist that can be easily implemented and used to flag problems with quality of care. Patient acceptability and satisfaction indicators can capture some aspects of quality. However, while easy to compile and reasonably affordable, they are subjective in nature [18], suffer from recall constraints [19] and are limited by the difficulty with clinical assessments [16]. Clinical vignettes are useful for measuring provider knowledge, but this is not a good indicator of provider effort since evidence suggests that knowledge and practice diverge [18,19,20,21]. Direct provider observation—while simple to execute—is subject to the Hawthorne effect [16]. Clinical audits of patient files have their own shortcomings and are often reliant on small samples [22,23,24]. Routinely captured health outputs—such as TB success and cure rates—are influenced by both demand and supply factors which make it difficult to interpret facility-level changes in such indicators.

The Standardised Patient (SP) method is an attempt to seek more accurate and reliable measurement of the clinical aspect of a healthcare interaction [15,25,26,27,28,29]. Although this method is regarded as a gold standard for measuring quality of care [30] it also suffers from weaknesses such as only being feasible for easily simulated conditions; the possibility of exposing SPs to hazardous environments; its limitation to contexts where ‘walk-ins’ are acceptable; and, the general dislike of the method by healthcare providers [16]. However, used alongside existing indicators, data derived from the SP method can offer a more comprehensive understanding of quality of care compared to alternative methods.

The purpose of this study is to objectively measure the quality of TB screening at metro-district primary healthcare (PHC) facilities in South Africa using the SP method. This is the first multi-district SP study in South Africa. The method allows researchers to observe how PHC providers identify, test and advise presumptive TB patients, and whether this aligns with clinical protocols and best practice.

## 2. Methods

### 2.1. Sampling and Setting

The study was conducted at 43% of PHC facilities in the Cape Town metropolitan district (Western Cape Province) and 26% in Buffalo City (Eastern Cape Province) in South Africa. It was assumed that urban facilities in the rest of the country would be similar. Furthermore, PHC expenditure per capita (uninsured) was similar in the districts for the 2015/2016 financial year—R1230 in Buffalo City and R1117 in Cape Town [31]. In total, 39 PHC facilities were covered, 19 in Cape Town (one facility was excluded due to access issues) and 20 in Buffalo City.

The 2015 TB (all types) incidence rates for both study districts exceeded the national average of 520 cases per 100,000, with Cape Town reporting a rate of 596 and Buffalo City a slightly higher rate of 743 [32]. For the same year, the known TB/HIV co-infection rate was 44.6% in Cape Town and 45.7% in Buffalo City [31].

Both Cape Town and Buffalo City PHC facilities implemented TB screening protocols that complied—at the very minimum—with the South African National TB Management Guidelines (SANTMG) of 2014 [32]. These guidelines formed the basis of the TB instruments (discussed in more detail below under Section 2.3.1).

### 2.2. The SP Method

The SP approach involved sending a covert fieldworker (SP) to a healthcare facility where he/she presented with an opening statement describing a set of pre-determined symptoms (TB symptoms in this case) that would predictably map to a set of clinical questions, examinations and tests. Closely approximating a real-life patient required rigorous training and precise instructions to the SP. The eight recruited SPs—four from each province—were trained according to a script with standard questions likely to be asked at a TB screening. Their responses were standardised and designed to avoid invasive examinations. The SPs were trained to only supply information in response to questions asked by the healthcare worker, with explicit instructions not to voluntarily disclose any additional information. The SPs presented using their own identities to reduce the likelihood of being detected.

Healthcare providers were unaware that the SP was not a real patient. This provided an opportunity to observe the healthcare worker’s actions in a natural environment. After the interaction, the SP captured details about the facility visit on a score sheet (discussed in more detail under Section 2.3.1). High reliability recall of SPs was demonstrated in a validation study that compared data collected through exit interviews with SPs post interaction with data collected through voice recordings of the SP-provider interaction [15]. While the validation study provides confirmation about the reliability of the method, we acknowledge that this would vary from case to case, similar to standard surveys and fieldwork. Due to ethical issues with recording nurse interactions with patients, it is not feasible to use voice recordings to validate SP recall in each SP study. In lieu of voice recordings, we employed quality control mechanisms and scrutinised SP motivation and recall prior to recruitment.

Comparing the clinical treatment received by the SP to clinical practice and protocols enabled researchers to measure clinical quality at the facility level. The rationale was to understand whether the healthcare worker asked the correct questions and conducted the required examinations and tests, measured according to standardised protocols and practice.

SP work in India [25] has shown that it provides reliable quality findings that do not necessarily correlate with and follow patterns of alternative facility-level measures such as patient satisfaction or clinical knowledge. The method has also been validated in other low- and middle-income settings including Kenya and China [25,27,33,34].

In TB research, SP studies are increasingly recognised as helping to identify and monitor deficiencies in health systems such as provider behaviour that may compromise the diagnostic evaluation [7,11].

### 2.3. Instruments and Data Collection

#### 2.3.1. TB Screening Instruments

The SP’s interaction with the healthcare provider was guided by a carefully developed script and documented using a comprehensive score sheet. Scripts and score sheets were developed based on publicly available instruments from an international SP manual [35], local TB screening guidelines (the SANTMG [32], Primary Care 101 [36], Practical Approach to Care Kit (PACK) Adult [37] and best practices. The score sheet made provision for capturing the most essential clinical components of the interaction first to minimise recall bias.

The TB screening instruments were reviewed by various external stakeholders in the respective study districts: clinical experts, PHC providers, public health specialists and programme managers (Table S1). They were then piloted at five PHC facilities in Cape Town in 2015 (*n* = 14). Pilot findings assisted us with refinement of the instruments which were sent out to relevant external stakeholders for a second round of review.

#### 2.3.2. Data Collection

The full roll-out of the project commenced at various stages during 2016 and early 2017, taking six months to complete. A sample size of 156 patient-facility interactions was anticipated (Figure S1) given that a total of 39 facilities would be visited by four SPs. Each SP visited study facilities in his or her province. A breakdown of SP characteristics can be found in Table S2. 

All SPs were provided with official letters which they could use in the event of being exposed as a covert fieldworker. The letter explained that the SP was part of a study endorsed by the respective departments of health. None of the SPs were detected during the study.

TB score sheets were completed by the SP in privacy immediately after leaving the facility to minimise recall lapses and biases. To monitor data capturing quality and reliability, a fieldwork manager conducted bi-weekly debriefing sessions with SPs where TB score sheets were audited.

After taking failed visits and ‘home facilities’ into account, the sample size was reduced to 143 interactions (Figure S1). To maintain the study integrity and the privacy of the SP, SPs were not allowed to visit any of the facilities they used regularly (also known as ‘home facility’). Failed SP visits were defined as an SP not entering the facility or being turned away from the facility at any point before being seen by a healthcare worker.

The study collected and aggregated anonymised data (Data S1 and Data S2). Where serious facility-level problems were detected, provincial health authorities were alerted.

### 2.4. Statistical Analysis

Clinical data derived from the TB instrument are presented as binary variables. We focus on variables that are included in TB screening protocols and best practices, and use uni- and bivariate analysis of these variables to describe the levels of the quality of TB screening. Data analyses were conducted at the facility visit level (and not at the individual healthcare provider level). Statistical analyses were performed using STATA (version 14.02, StataCorp LLC, College Station, TX, USA) software (Do File S1).

### 2.5. Ethical Considerations and Approval

Ethical concerns for the SP method include the risks that the SP is exposed to as well as the issue of concealment. Risks to the SP were minimised during training and preparation, as well as by intentionally designing scenarios that did not involve invasive examinations (such as TB screening). Scripts also included excuses that the SP could use to prevent further examination should they need to exit an interaction.

The concealment involved in the SP method differentiates this work from traditional surveys. Concealed research—also referred to as covert research—is regarded as permissible under certain circumstances if it is integral to the nature of the research being conducted, i.e., non-concealment will undermine the purpose of the research [38]. This study was granted ethical clearance from the Research Ethics Committee for Human Research (Humanities) at Stellenbosch University, South Africa (HS1096/2014-REC).

## 3. Results

Summary statistics for the variables of interest were generated (Table S4). Results are reported as a percentage, followed by the absolute value and 95% confidence interval in brackets. Variables of interest were stratified by SP, facility (Table S5), gender and age (Table S6). No consistent pattern of significant variation in means emerged, except across SPs—and to a lesser degree—across facilities.

### 3.1. Case Description and Management

The case description for each SP was presumed TB with more than two weeks of coughing, fever and weight loss. Having the SPs present in this manner allowed us to observe how PHC providers identify, test and advise presumptive TB patients.

The failed visit rate captures the proportion of unsuccessful facility visits where the SP left the facility without consulting a provider. This rate makes provision for excluding cases where the SP could not visit a facility because it was their local or ‘home facility’. Overall, the failed visit rate was 6% or nine out of a potential 152 patient-provider interactions (Table S3).

For the 143 analysable interactions SPs presented with the following opening statement: ‘I have been coughing a lot recently’. Programme managers and clinicians advised against adding ‘for two weeks’ because this could appear contrived or rehearsed and not aligned with how patients who are concerned about their cough would normally present at facilities. If they were not triaged or identified for TB screening, SPs were trained to add: ‘I think I may have TB’. We required SPs to track whether they mentioned TB explicitly in their opening statement. More than 90% (91%; 124; 0.85–0.95) of SPs were triaged for TB screening without the need to mention TB.

### 3.2. Adequate Case Management

Based on a review of guidelines and protocols, and consultations with clinical experts and programme managers, we defined a minimum level of case management for TB screening as a sputum test and an offer of a HIV test. Applying this definition, we find that 43% (61; 0.35–0.51) of SPs were correctly managed. This result is driven mainly by a low rate of HIV tests offered, since sputum tests were conducted at 84% (120; 0.77–0.89) of visits while HIV tests were offered at only 47% (67; 0.39–0.55). SPs were asked about household TB contacts in 54% (77; 0.46–0.62) of cases.

Using the SANTMG benchmark of checking for four essential TB symptoms: coughing for more than two weeks, weight loss, night sweats, and fever for more than two weeks, the overall finding shows that just over half (55%) of the essential history checklist questions were asked. The prevalence of the questions asked was: cough duration (80%; 114; 0.73–0.86), night sweats (59%; 84; 0.50–0.67) weight loss (55%; 78; 0.46–0.63) and duration of fever (25%; 36; 0.19–0.33).

### 3.3. Medical Examinations

Blood pressure and weight measurements were performed most frequently (43% (61; 0.35–0.52) and 38% (54; 0.31–0.47), respectively) at SP TB screenings. Pulses were checked the least frequently at 13% (18; 0.08–0.20) of interactions. Temperatures were taken at 16% (22; 0.10–0.27) of interactions overall and in 22% (8; 0.11–0.39) of cases where the SPs were asked whether they had a fever (the standardised response was yes).

### 3.4. Dispensing of Antibiotics

For interactions where no temperatures were taken, antibiotics were prescribed 8% (10; 0.05–0.15) of the time (10 out of 119 cases). Amoxycillin was prescribed for most of these cases (eight), while penicillin was prescribed for one case and a trimethoprim and sulfamethoxazole combination antibiotic for another.

### 3.5. Access to Surgical Masks

SPs had access to surgical masks in 48% (69; 0.40–0.57) of cases.

### 3.6. Follow-Up

Fifteen per cent (15; 0.09–0.23) of SPs did not receive any verbal or written communication about returning to collect their TB test results. Only 28% (33; 0.20–0.37) reported that nurses explained the importance of returning for their TB test results.

## 4. Discussion

To be considered of acceptable quality, TB care needs to comply with (international) standards [7]. For South Africa, TB screening protocols used at a PHC level comply with the SANTMG which is derived from the World Health Organization’s TB guidelines. The discussion highlights some of the missed opportunities in the current implementation of TB screening protocols which were identified using the SP method.

While an overall failed visit rate of 6% may seem low at first glance, this translated into nine of the SPs not having the opportunity to be screened for TB (Table S3). This represents a loss of presumptive TB patients from the TB care cascade at the earliest point. In this study, SPs were not given a waiting time in terms of how long they should wait before leaving, i.e. SPs could only leave the facility when instructed to do so by staff at the facility. Often no reason was provided when SPs were told to leave the facility, other times they were informed it was due to staff constraints, e.g., a nurse being on sick leave. Patients who have experienced failed visits at a PHC facility may also become discouraged and could be reluctant to return. Systems need to be developed to ensure that presumptive TB patients are prioritised, even when facilities are short-staffed.

Given the prioritisation of HIV and TB care in South Africa over the past two decades through additional funding and training, it is disappointing to see that in only 43% of cases did the public healthcare system meet the minimum threshold for adequate case management. This was driven mainly by a lower than expected rate of offering HIV tests (47% of cases) which is concerning given the high TB/HIV co-infection rate in South Africa (57% in 2015 [31]) and the resources that have been invested in training, awareness and fighting stigma. It is also worrisome because the level of sensitivity of TB test results using Xpert**^®^**, the recommended TB diagnostic tool in South Africa, is influenced by HIV status [39]. False-negative TB test results carry costs for the individual and perpetuate the spread of infectious TB. Even though sputum tests were conducted often (84% of visits), there is room for improvement.

Besides people who are HIV positive, the SANTMG recommends that a high index of suspicion is required for patients who have been in contact with a person with confirmed TB. From this perspective, enquiring whether anyone in the household had confirmed TB was viewed as important by TB control programme managers. Despite this, SPs were asked about household TB contacts in only 54% of cases.

There are many potential reasons for deviations from protocols including inadequate training, lack of knowledge, deficiencies in monitoring and evaluation [40,41] and, more broadly, a lack of clinical governance [42,43,44,45]. Unfortunately, due to our limited sample, we cannot test the plausibility of rival explanations for poor protocol compliance.

Checking for the four main symptoms of pulmonary TB is essential in a clinical interaction since every patient with a positive TB symptom should be tested for TB, according to the SANTMG. The overall finding shows that just over half of the essential history checklist questions were asked. Similarly, physical examinations linked to TB screening were conducted at less than 50% of SP interactions. Cough duration emerged as the most frequently asked essential history checklist question, which implies that healthcare providers relied almost exclusively on this symptom when determining whether the patient requires a sputum test or not.

In less than 10% of the interactions SPs had to provide the additional statement of ‘I think I may have TB’ before being triaged or identified for TB screening. This reinforces a hypothesis that persistent coughing, the opening statement of the SP, is top of mind as a TB symptom and elicits the appropriate referral within the facility.

A worrisome finding emerged: 8% of SP interactions where no temperatures were taken resulted in the prescription of antibiotics. Considering the local antibiotic algorithm and TB screening protocol, there was no indication for antibiotics to be administered. Practices such as this may lead to an increase in drug-resistant bacteria in South Africa. Further research is needed to understand why this practice occurs. It may be a form of empirical antibiotic treatment that is practiced in certain countries [15], though the South African TB protocol does not allow for empirical antibiotic treatment unless the patient has a temperature greater than, or equal to, 38 degrees Celsius [36]. Irrespective of the rationale, the risks associated with this practice are high and, at this preliminary stage, should be considered unsafe.

Providing surgical masks has been deemed a best practice by clinical experts and programme managers and is considered an infection-control measure. SPs had access to a surgical mask at less than half of interactions (48%). Making surgical masks more readily available to all patients entering facilities is a simple and pragmatic way of reducing the risk of droplet infection [46,47].

Lack of information-sharing, particularly in terms of continuity of care, emerged during the interactions: approximately two out of ten SPs were not informed about returning to the facility for their TB test results. Even when cases were managed correctly, very little effort was made to explain the importance of returning for TB test results. This was explained at less than a third of interactions (28%). The overall trend suggests low effort exerted in communicating valuable information to the patient regarding the dangers of untreated TB and the need to start treatment as soon as possible.

This suggests that a diagnostic gap exists with some presumptive TB patients leaving facilities without being tested for TB; others leaving the facility without knowing that they need to return for results and why; and, some TB test results may come back as a false-negatives. These findings about deficiencies in diagnostic protocol compliance add to the literature analysing the weaknesses in the health system and can provide useful feedback to national and provincial governments.

We argue, based on this study and the relevant studies cited, that the care provided for TB screening in South Africa is not as effective as it could be given its resources. The multiple gaps in protocol adherence are a concern that needs to be acknowledged and addressed because they contribute to TB deaths and disappointing TB outcomes. Protocols and best practices may need to be interrogated to ensure that their most important elements are prioritised, allowing for more pragmatic implementation in high-burden settings. Ensuring that basic and important steps in the TB screening protocol are adhered to may prevent the loss of presumptive TB patients in the healthcare system.

The increasing popularity of the SP method in measuring the quality of healthcare is supported by the findings of this study which was able to identify deficiencies in the health system, specifically provider behaviour that compromised the diagnostic evaluation of TB patients. The method has been used as a complementary but ad hoc monitoring tool in other countries [28].

## 5. Conclusions

Use of the SP method to measure the quality of TB screening—the first endeavour of its kind in South Africa—shows evidence of gaps in clinical practice, signalling missed opportunities for diagnosis. Early and reliable detection of sub-optimal quality of TB care is instrumental in decreasing TB-related morbidity and mortality, highlighting the usefulness of the SP method. The study findings provide the foundation for further quality and quality improvement in the management of TB, particularly in high-burden countries such as South Africa.

## Supplementary Materials

The following are available online at http://www.mdpi.com/1660-4601/15/4/729/s1, Data S1: Dataset saved in STATA, Data S2: Dataset saved in excel, Do File S1: STATA commands for analysis, Figure S1: Exclusions from standardised patient-facility interactions, Table S1: List of external reviewers of TB screening instruments, Table S2: Standardised patient characteristics, Table S3: Failed visit rate of standardised patients, Table S4: Summary statistics of variables of interest, Table S5: One-way ANOVA of variables of interest by standardised patient and facility, Table S6: One-way ANOVA of variables of interest by gender and age.
