# Supplementary material for: Measuring Quality Gaps in TB Screening in South Africa Using Standardised Patient Analysis
Source: Int J Environ Res Public Health. 2018 Apr 12;15(4):729. doi: 10.3390/ijerph15040729 (PMC5923771; doi:10.3390/ijerph15040729)
Supplement: Supplementary file 1 [file ijerph-15-00729-s001.zip › Supplementary figure (Figure S1) and tables (Table S1 to Table S6).pdf]

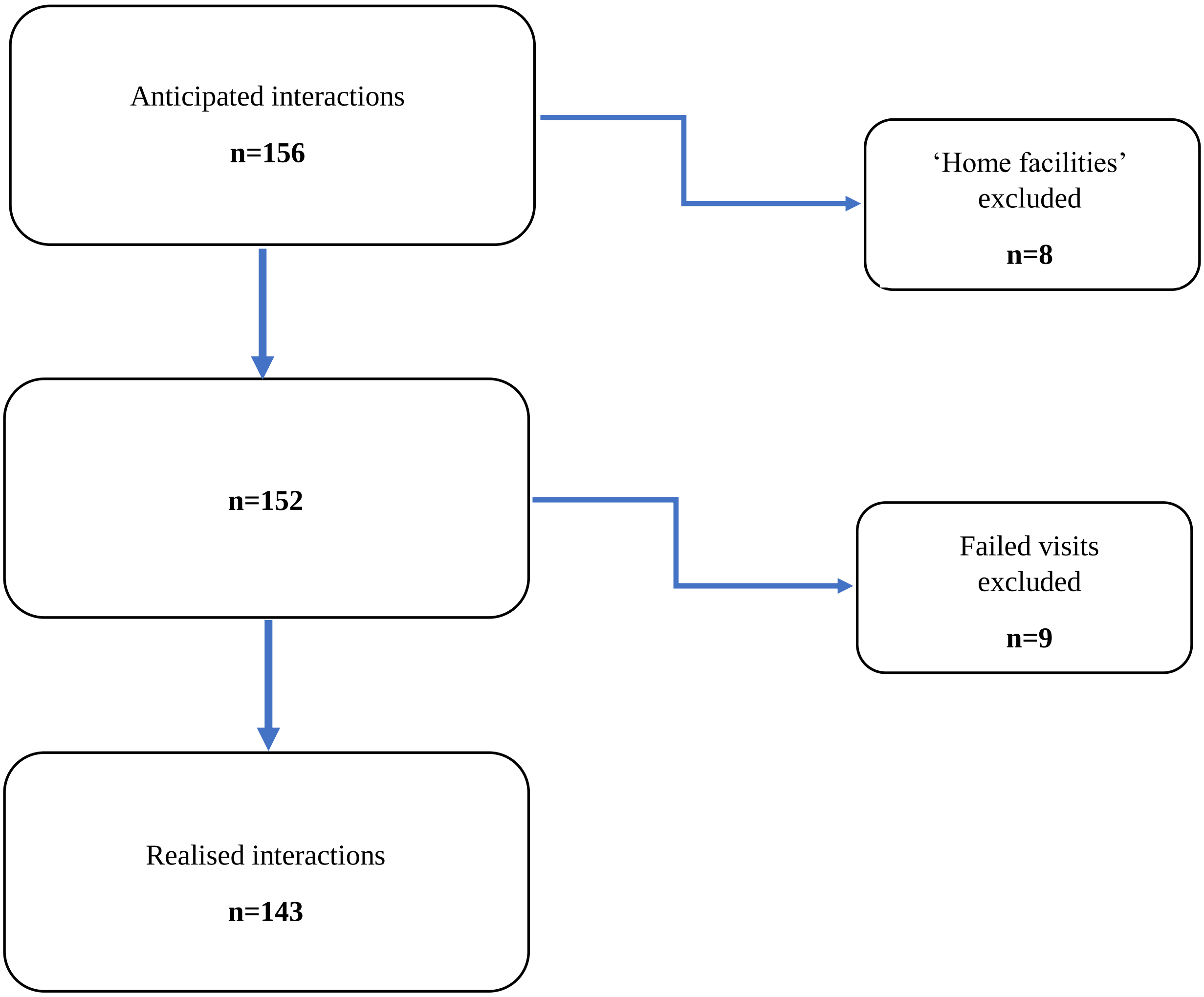

Figure S1: Exclusions from standardised patient-facility interactions

'Home facility' refers to a primary healthcare facility that the SP used regularly. Failed visits were defined as an SP not entering the facility or being turned away from the facility at any point before being seen by a healthcare worker.

Table S1: List of external reviewers of TB screening instruments

Department, Institution	Job Title	Inputs
Health Impact Assessment Unit, Department of Health, Western Cape Government	Public Health Specialist (1)	Provided referrals to relevant stakeholders for review of instruments.
	Public Health Specialist (2)	Provided the relevant TB guidelines. Reviewed early versions of the instruments.
Desmond Tutu TB Centre, Stellenbosch University	Research Clinician (1)	Provided clinical expertise when drafting instruments. Reviewed instruments.
	Research Clinician (2)	Provided clinical expertise when drafting instruments. Reviewed instruments.
Facility-based Programmes, Department of Health, Western Cape Government	Programme Manager	Provided insights about integrated approach to primary healthcare assessment. Ensured that instruments contained integrated clinical questions.
HAST (HIV & AIDS, STI and TB) Directorate: TB Prevention and Management, Department of Health, Western Cape Government	Deputy Director	Provided insights about the current management of presumed TB patients at primary healthcare facilities. Reviewed instruments and provided inputs.
	Assistant Deputy Director	Provided insights about the current management of presumed TB patients at primary healthcare facilities. Reviewed instruments.
Integrated Strategic Management, Eastern Cape Government Department of Health	Chief Director	Provided referrals to relevant stakeholders for review of instruments.
TB Directorate, Department of Health, Eastern Cape Government	Provincial Programme Manager	Provided insights about the current management of presumed TB patients at primary healthcare facilities. Reviewed instruments and provided inputs.

Instruments include both the TB screening script and score sheet.

Table S2: Standardised patient characteristics (n=8)

**Characteristic**		**n (%)**
**Gender**	Male	4 (50)
	Female	4 (50)
**Age**	<30 years	4 (50)
	≥30 years	4 (50)
**Race**	Black	6 (75)
	Coloured	2 (25)
**Education**	< Matric	1 (12.5)
	Matric	3 (37.5)
	> Matric	4 (50)

Matric is the final year of secondary schooling, i.e. 12 years of education completed and passed.

In South Africa there are four main racial classifications – Black, Coloured, White and Indian.

Table S3: Failed visit rate of standardised patients

	**Total**
**Anticipated SP interactions**	n=156
**Facility not visited since SP's 'home facility'**	n=4
**Failed visits**	n=9
**Realised SP interactions**	n=143
**Failed visit rate**	0.06

'Home facility' refers to a primary healthcare facility that the SP used regularly.

Failed visits were defined as an SP not entering the facility or being turned away from the facility at any point before being seen by a healthcare worker.

Table S4: Summary statistics of variables of interest

Variable	Observations	Mean	Std. Dev.	Min	Max
**Adequately managed**	143	.4265734	.4963176	0	1
**TB test conducted**	143	.8391608	.3686739	0	1
**HIV test offered**	143	.4685315	.5007627	0	1
**Asked about household TB contacts**	143	.5384615	.5002708	0	1
**Opening statement only 'coughing a lot'**	136	.9117647	.2846854	0	1
**Asked about cough duration**	142	.8028169	.3992801	0	1
**Asked about night sweats**	143	.5874126	.4940302	0	1
**Asked about weight loss**	143	.5454545	.4996798	0	1
**Asked about fever duration**	143	.2517483	.4355429	0	1
**Blood pressure measured**	141	.4326241	.4972060	0	1
**Weight measured**	141	.3829787	.4878462	0	1
**Pulse checked**	140	.1285714	.3359269	0	1
**Temperature taken**	141	.1560284	.3641759	0	1
**Temperature taken if asked about fever duration**	36	.2222222	.421637	0	1
**Antibiotics prescribed without taking temperature**	119	.0840336	.2786113	0	1
**Access to surgical mask**	143	.4825175	.5014507	0	1
**Return for TB test results communicated**	103	.8543689	.3544608	0	1
**Explained importance of returning for TB test results**	119	.2773109	.4495642	0	1

Table S5: One-way ANOVA of variables of interest by standardised patient and facility

Variable	Between standardised patients			Between facilities		
	Lowest mean	Highest mean	Prob > F	Lowest mean	Highest mean	Prob > F
**Adequately managed**	.05263158	1	0.0000	0	1	0.0000
**TB test conducted**	.36842105	1	0.0000	.5	1	0.5284
**HIV test offered**	.05263158	1	0.0001	0	1	0.0000
**Asked about household TB contacts**	0	.89473684	0.0002	0	1	0.5665
**Opening statement only 'coughing a lot'**	.68421053	1	0.0004	.5	1	0.7803
**Asked about cough duration**	.47368421	1	0.0020	.33333333	1	0.5941
**Asked about night sweats**	.10526316	1	0.0000	.25	1	0.3508
**Asked about weight loss**	.31578947	1	0.0558	0	1	0.1764
**Asked about fever duration**	0	.52631579	0.0004	0	1	0.1751
**Blood pressure measured**	0	.8	0.0524	0	1	0.0040
**Weight measured**	0	.52631579	0.7884	0	1	0.0000
**Pulse checked**	0	.53333333	0.0000	0	.5	0.8192
**Temperature taken**	0	.33333333	0.3495	0	.75	0.0004
**Temperature taken if asked about fever duration**	0	.66666667	0.3638	0	1	0.7215
**Antibiotics prescribed without taking temperature**	0	.47058824	0.0000	0	.66666667	0.6943
**Access to surgical mask**	.6875	1	0.0018	0	1	0.0005
**Return for TB test results communicated**	.5625	1	0.0059	0	1	0.0691
**Explained importance of returning for TB test results**	0	.69230769	0.0030	0	1	0.0031

Standardised patient: n=8; Facility: n=39.

Table S6: One-way ANOVA of variables of interest by gender and age

Variable	Between genders			Between age categories		
	Men mean	Women mean	Prob > F	≤ 30 years mean	> 30 years mean	Prob > F
**Adequately managed**	.43478261	.41891892	0.8493	.375	.50909091	0.1163
**TB test conducted**	.92753623	.75675676	0.0052	.80681818	.89090909	0.1855
**HIV test offered**	.44927536	.48648649	0.6586	.40909091	.56363636	0.0725
**Asked about household TB contacts**	.71014493	.37837838	0.0000	.67045455	.32727273	0.0000
**Opening statement only 'coughing a lot'**	.92424242	.9	0.6215	.87058824	.98039216	0.0289
**Asked about cough duration**	.82608696	.78082192	0.5015	.86363636	.7037037	0.0200
**Asked about night sweats**	.68115942	.5	0.0279	.70454545	.4	0.0003
**Asked about weight loss**	.66666667	.43243243	0.0047	.59090909	.47272727	0.1697
**Asked about fever duration**	.23188406	.27027027	0.6002	.29545455	.18181818	0.1295
**Blood pressure measured**	.46376812	.40277778	0.4685	.47727273	.35849057	0.1703
**Weight measured**	.36231884	.40277778	0.6242	.35227273	.43396226	0.3373
**Pulse checked**	.15942029	.09859155	0.2857	.18181818	.03846154	0.0142
**Temperature taken**	.17391304	.13888889	0.5699	.15909091	.1509434	0.8982
**Temperature taken if asked about fever duration**	.25	.2	0.7292	.19230769	.3	0.5004
**Antibiotics prescribed without taking temperature**	.03508772	.12903226	0.0659	.13513514	0	0.0097
**Access to surgical mask**	.52173913	.44594595	0.3683	.5	.45454545	0.5997
**Return for TB test results communicated**	.8852459	.80952381	0.2889	.88235294	.8	0.2661
**Explained importance of returning for TB test results**	.21875	.34545455	0.1258	.21126761	.375	0.0509

Men: n=4; Women: n=4; ≤ 30 years: n=5; > 30 years: n=3.
